# Knowledge deficit and fear of COVID-19 among higher education students during the first wave of the pandemic and implications for public health: a multi-country cross-sectional survey

**DOI:** 10.1186/s12889-022-13511-3

**Published:** 2022-06-07

**Authors:** Neamin M. Berhe, Sarah Van de Velde, Fatemeh Rabiee-Khan, Claudia van der Heijde, Peter Vonk, Veerle Buffel, Edwin Wouters, Guido Van Hal

**Affiliations:** 1 Department of Epidemiology, Medicine & Health Sciences Faculty, University of Antwerp, Antwerp, Belgium; 2 Centre for Population, Family and Health, Department of Sociology, University of Antwerp, Antwerp, Belgium; 3 Faculty of Health, Education & Life Sciences, Birmingham City University, Birmingham, UK; 4 Department of Student Health Services, University of Amsterdam, Amsterdam, Netherlands; 5 Department of Social Epidemiology and Health Policy, University of Antwerp, Antwerp, Belgium

**Keywords:** COVID-19, Knowledge, Fear, Multi-country, Students, Mixed-effect logistic regression

## Abstract

**Background:**

Public health measures such as physical distancing and distance learning have been implemented during the COVID-19 pandemic. COVID-19 related knowledge deficit can increase fear that leads to negative mental health and COVID-19, especially among adolescents. Therefore, our study aimed to assess COVID-19 related knowledge deficit and its association with fear among higher education (HE) students during the first wave of COVID-19.

**Methods:**

A cross-sectional survey, COVID-19 International Students Well-being Study (C-19 ISWS) was conducted in 133 Higher Education Institutions (HEIs) in 26 countries between April 27 and July 7, 2020. A stratified convenience sampling technique was used. Descriptive, bivariate, mixed-effect logistic regression analyses were conducted using R software.

**Results:**

Out of 127,362 respondents, 72.1% were female, and 76.5% did not report a previous history of confirmed COVID-19. The majority of those without the previous infection 81,645 (83.7%) were from 21 European countries while the rest 15,850 (16.3%) were from 5 non-European countries. The most frequent correct response to COVID-19 related knowledge questions among respondents was having the virus without having symptoms (94.3%). Compared to participants with good knowledge, the odds of being afraid of acquiring SARS-COV-2 infection among those with poor knowledge was 1.05 (95%CI:1.03,1.08) and the odds of being afraid of contracting severe COVID-19 was 1.36 (95%CI:1.31,1.40).

**Conclusion:**

COVID-19 related knowledge was independently associated with both fear of acquiring SARS-COV-2 infection as well as contracting severe COVID-19. Our findings will serve as a basis for public health response for both the current and similar future pandemics by highlighting the need for addressing the COVID-19 knowledge deficit to fight the infodemic and prevent negative mental health outcomes.

**Supplementary Information:**

The online version contains supplementary material available at 10.1186/s12889-022-13511-3.

## Introduction

Coronavirus disease (COVID-19) generated a world-wide public health emergency which the World health organization (WHO) declared a pandemic on 11th of March 2020 [1]. In response, governments have introduced a range of public health measures aimed at limiting the spread of infection alongside the provision of treatment for those who fall ill. However, the effectiveness of these measures is largely dependent on the cooperation of the population in general and specific risk groups in particular [2]. The extent to which public health measure is adhered to vastly depends on disease-related knowledge of the individual. COVID-19 is the first pandemic in history in which technology and social media are being used on a massive scale to keep people safe & informed which has also unfortunately enabled and amplified an infodemic [3, 4]. WHO has described infodemic as an overabundance of information including deliberate attempts to disseminate wrong information to undermine the public health response [3, 4]. Incorrect and misleading information including knowledge deficit can lead to anxiety, rejection of measures in place, depression and negative COVID-19 outcomes [2, 5–7]. A previous study carried out on COVID-19 patients found a negative association between level of education and worry for family and others contracting the disease [2] while other studies have found a positive association between knowledge on COVID-19 and level of education [7–9]. A study has also found COVID-19 related knowledge to be positively correlated with both risk perception and preventative behaviours [10]. An assessment of COVID-19 related knowledge and its association with fear about COVID-19 is especially important in young adults as misinformation about the vulnerability of this age group to COVID-19 has been highlighted by various influential groups [11]. As the pandemic grew, health institutions and relevant stakeholders were not only dealing with virus but also with the infodemic mainly spread through social media platforms that negatively impacted the response to COVID-19 [12]. Problematic social media use and its negative consequences including misinformation are rampant especially among this age group [13]. Additionally, higher education (HE) students have high mobility and contribute significantly to the spread of the infectious COVID-19 [14] which may also have a substantial impact on their social and mental wellbeing with possible long-term consequences [5, 14, 15]. Mental health issue among HE students is a leading public health concern which have only been exacerbated by the pandemic mainly due to fear among other driving factors [16]. Another recent study have also found that HE students are vulnerable for either developing or experiencing exacerbated pre-existing depressive symptoms during the COVID-19 pandemic [17]. However, No study assessing COVID-19 related knowledge and fear about COVID-19 among HE students has to our knowledge been published so far. Such a study would help in addressing this gap and have the potential to increase adherence to public health measures [5, 7, 15]. It will also help develop appropriate public health interventions to address HE students’ social and mental health issues especially during a pandemic. Therefore, this study aimed to assess COVID-19 related knowledge deficit among HE students in 26 countries and its association with fear about both Severe COVID-19 and SARS-COV-2 (severe acute respiratory syndrome coronavirus 2) infection. In this study, we hypothesized that COVID-19 knowledge deficit among HE students can lead to increased fear of acquiring Severe COVID-19 and SARS-COV-2 infection.

## Methods

### Study design and setting

Study design was cross-sectional, using Qualtrics online survey software (Qualtrics, Provo, UT). An online questionnaire was the only feasible method of data collection during the pandemic. Data were collected between 27 April and 7 July, 2020. 133 HEIs in 26 countries agreed to take part and participated in COVID-19 International students’ well-being study (C-19 ISWS) [18].

### Sampling technique and characteristics of participants

The C-19 ISWS applied a stratified convenience sampling design. HEIs were selected from a total of 26 countries in western, central, eastern, northern and southern Europe including Canada, Israel, South Africa, Turkey and USA (Table 1). Students aged 18 years or above were eligible to take part irrespective of their level of study. Those who had been officially diagnosed with COVID-19 were excluded as the aim was to assess fear about infection and not re-infection. A consortium partner was appointed in each HEI to take responsibility for seeking ethical approval. The consortium partners were also responsible for distributing a link to the survey within their HEI via e-mail to all students. Alternative recruitment methods were; student-specific social media platforms, and newspapers [18]. This link led to information about the study and the consent form. Students were informed that participation was voluntary and of their right to withdrawal. No identifying personal details were collected to ensure participant anonymity. Participants were asked to indicate consent by ticking a box before starting the survey. A reminder e-mail was sent after 1 week.

**Table 1:** Characteristics of study participants by country

Country	Age (in years)			Total n(%)	Gender		
	17–20 n(%)	21–24 n(%)	> = 25 n(%)		Male n(%)	Female n(%)	X n(%)
Belgium	9234(9.5)	9110(9.3)	2753(2.8)	21,099(21.6)	5493(5.6)	15,508(15.9)	98(0.1)
Canada	768(0.8)	1414(1.5)	1904(2.0)	4096(4.2)	1221(1.3)	2826(2.9)	49(0.1)
Czech	1382(1.4)	3853(4.0)	1792(1.8)	7027(7.2)	1943(2.0)	5050(5.2)	34(00.1)
Denmark	104(0.1)	995(1.0)	1175(1.2)	2275(2.3)	487(0.5)	1774(1.8)	14(< 0.01)
Finland	101(0.1)	434(0.4)	545(0.6)	1080(1.1)	224(0.2)	839(0.9)	17(< 0.01)
France	2202(2.3)	1650(1.7)	499(0.5)	4351(4.4)	1194(1.2)	3124(3.2)	33(< 0.01)
Germany	867(0.9)	2269(2.3)	1833(1.9)	4972(5.0)	1467(1.5)	3449(3.5)	56(0.01)
Greece	183(0.2)	300(0.3)	115(0.1)	598(0.6)	175(0.2)	419(0.4)	4(< 0.01)
Hungary	614(0.6)	1158(1.2)	764(0.8)	2537(2.6)	795(0.8)	1730(1.8)	12(< 0.01)
Iceland	37(0.001)	132(0.1)	339(0.3)	514(0.5)	105(0.1)	403(0.4)	6(< 0.01)
Israel	52(0.1)	153(0.2)	210(0.2)	416(0.4)	99(0.1)	316(0.3)	1(< 0.01)
Italy	2082(2.1)	5017(5.1)	2266(2.3)	9374(9.6)	2362(2.4)	6960(7.1)	52(0.1)
Netherland	3556(3.6)	5790(5.9)	2102(2.2)	11,450(11.7)	3330(3.4)	8038(8.2)	82(0.1)
Norway	297(0.3)	1305(1.3)	1582(1.6)	3186(3.2)	1012(1.0)	2160(2.2)	14(< 0.01)
Portugal	359(0.4)	324(0.3)	181(0.2)	865(0.8)	138(0.1)	723(0.7)	4(< 0.01)
Romania	202(0.2)	409(0.4)	59(0.1)	670(0.6)	140(0.1)	530(0.5)	0(< 0.01)
Russia	1561(1.6)	1033(1.1)	120(0.1)	2714(2.7)	642(0.7)	2072(2.1)	0(< 0.01)
Slovakia	194(0.2)	481(0.5)	159(0.2)	835(0.8)	237(0.2)	596(0.6)	2(< 0.01)
South Africa	508(0.5)	405(0.4)	221(0.2)	1134(1.1)	275(0.3)	849(0.9)	10(< 0.01)
Spain	274(0.3)	398(0.4)	234(0.2)	908(0.9)	267(0.3)	630(0.6)	11(< 0.01)
Sweden	136(0.1)	494(0.5)	625(0.6)	1262(1.2)	428(0.4)	824(0.8)	10(< 0.01)
Switzerland	559(0.6)	1937(2.0)	1109(1.1)	3607(3.6)	941(1.0)	2625(2.7)	41(< 0.01)
Turkey	3035(3.1)	4808(4.9)	2197(2.3)	10,043(10.3)	2994(3.1)	6994(7.2)	55(0.1)
UK	634(0.7)	727(0.7)	670(0.7)	2039(2.0)	436(0.4)	1588(1.6)	15(< 0.01)
USA	81(0.1)	61(0.1)	19(0.001)	161(0.1)	28(0.01)	128(0.1)	5(< 0.01)
Cyprus	104(0.1)	124(0.1)	54(0.1)	282(0.2)	71(0.1)	210(0.2)	1(< 0.01)
Total	29,126 (29.9)	44,781 (45.7)	23,527 (24.1)	97,495(100)	26,504(27.2)	70,365(72.1)	626(0.006)

### Data measurement/operational definition

*Fear about SARS-COV-2 infection (outcome variable 1)* referred to whether respondents are worried about having mild illness and/or asymptomatic infection of COVID-19 clinical manifestation [19]. It was measured using a scale ranging from 0 to 10. Zero indicated “not worried” and ten indicated “very worried” about SARS-COV-2 infection. The mean value was utilized to classify the variable into two categories: “afraid” and “unafraid” in this study. Respondents with a scale response of equal or less than 4.2 were classified as “unafraid”. Scores above 4.2 were classified as “afraid”. *Fear about Severe COVID-19 (outcome variable 2)* referred to whether respondents are worried about having severe and critical illness of COVID-19 clinical manifestation [19]. It was measured using a scale ranging from 0 to 10. Zero indicated “not worried” and ten indicated “very worried” about Severe COVID-19. The mean value was utilized to classify the variable into two categories: “afraid” and “unafraid”. Respondents with a scale response of equal or less than 3.1 were classified as “unafraid”. Scores above 3.1 were classified as “afraid”. Eight questions were used to capture *COVID-19 related knowledge deficit (exposure Variable)*. To describe respondents’ knowledge level, it was categorized as “Good” if more than five questions were answered correctly, and as “Poor” if they scored four or less. To assess association, mixed-effect logistic regression coefficients were used for each “correct” response and the mean values were used to categorize total knowledge score. Respondents’ knowledge level above the mean value were categorized as “Good”, and below or equal to the mean value as “Poor”. Socio-economic factors were considered potential confounders in our study: age, sex, educational level, field of study, financial resources, underlying medical conditions, personal networks, infection history, adherence to public health measures, risk perception, alcohol use, smoking, moderate and vigorous physical activity. Respondents were asked what these were like in the month before public health measures were introduced and what they were like in the week before completing the questionnaire. The difference between the two was used to calculate the degree of change. Satisfaction with timely government information was also measured.

### Data analysis

All analyses were carried out using the statistical package R (R Foundation for Statistical Computing©, version 3.5.3). Descriptive statistics on key characteristics of respondents and COVID-19 related knowledge across the 26 countries were displayed using tables and graphs. Polychloric correlation matrix for the eight COVID-19 related knowledge (categorical variables) was carried out to assess the need for conducting exploratory factor analysis (EFA). Missing data were handled using the multiple imputation chained equation (MICE) package in R. Due to some behavioural variables and sensitive questions there were missing data in the study (< 10%) in each variable. Variables were assumed to be missing at random (MAR). Results from the primary analysis conducted using the MICE package were compared with the complete case analysis as a sensitivity analysis. Fear about getting SARS-COV-2 infection, acquiring severe COVID-19, and COVID-19 related knowledge along with other independent variables using the DAG was included in the imputation model (total of 22 variables) (Supplementary: Figs. S1 & S2). Missing data for each variable was explored for patterns and the percentage of that was missing (Supplementary: Figs. S3 & S4). Bivariate analysis was used to assess the association between dependent and independent variables. Directed acyclic graph (DAG) with a priori assumption minimal sufficient adjustment set was used to introduce variables into the multivariable model [20]. Mixed-effect logistic regression model, accounting for possible random effects at country level, was implemented to assess the independent significant association between COVID-19 related knowledge and fear about SARS-COV-2 infection as well as fear about severe COVID-19. Bivariate, as well as mixed effect logistic regression analysis, were based on the pooled estimates from 20 imputed datasets, and *p*-values of < .05 were considered to be statistically significant. A seed value of 600 and using iteration of 20 was done. The complete case analysis was not different from the multiple imputed analyses. Both the estimate and the standard error of the variables were the same in both analyses.

## Results

### Characteristics of study participants

A total of 127,362 responses were received; 70,365 (72.1%) of participants were female. The majority of respondents 97,495 (76.5%) did not report a previous history of confirmed COVID-19 infection, of those 81,645 (83.7%) were from 21 European countries and the rest 15,850 (16.3%) were from 5 non-European countries. The highest number of respondents 21,099 (22%) were from Belgium, while the least number 161 (0.1%) were from USA. The majority of participants were aged 21–24 (45.7%) followed by under 21 years (29.9%) and above 24 years of age (24.1%) respectively (See Table 1, for detailed information).

Romania had the highest proportion of responses from health field students 641 (95.6%) and Cyprus had the lowest 20 (0.07%) (Supplementary: Fig. S5).

### COVID-19 related knowledge

COVID-19 related knowledge was “good” in 61,145 (62.7%) but only 5615 (5.7%) answered all the 8 COVID-19 related knowledge questions correctly, while the rest of participants were categorized as “Poor” COVID-19 related knowledge (Table 2).

**Table 2:** COVID-19 related knowledge responses

COVID-19 related Knowledge questions	Response			% of correct response
	True(%)	False(%)	Don’t know(%)	
The virus survives for days outside the body in open air. (Knowledge question A)	19,600(20.1)	57,195(58.6)^a^	18,338(18.8)	58.6
The virus survives for a week outside the body on a plastic surface. (Knowledge question B)	26,695(27.3)^a^	44,329(45.4)	23,859(24.4)	27.3
Most people who get COVID-19 get very ill. (Knowledge question C)	7003(7.1)	83,507(85.6)^a^	4743(4.8)	85.6
A possible vaccine will take around 12 to 18 months to produce(from the time of data collection)(Knowledge question D)	65,928(67.6)^a^	6099(6.2)	23,008(23.5)	67.6
Smokers who get COVID-19 are more likely to get severely ill than non-smokers.(Knowledge question E)	59,301(60.8)^a^	11,855(12.1)	23,913(24.5)	60.8
You can have the virus without any symptoms. (Knowledge question F)	91,966(94.3)^a^	1214(1.2)	2113(2.1)	94.3
On average, children get less ill from the virus than adults. (Knowledge question G)	78,052(80)^a^	7281(7.4)	9748(9.9)	80
Only elderly people die from COVID-19.(Knowledge question H)	1702(1.7)	91,783(94.1)^a^	1803(1.8)	94.1

^a^indicate the correct response for the respective COVID-19 related knowledge question

The most frequent correct response was to the question about having the virus without having symptoms (94.3%). While the least frequent correct response was the question about the duration of virus survival on plastic surfaces (27.3%).

Romania, Portugal and Finland had the highest respondents with “good” COVID-19 related knowledge with 89, 88.2 and 77.8%, respectively. Russia, South Africa and Israel had the lowest “good” scores on COVID-19 related knowledge with 31.8, 45.9 and 47.9% respectively (Supplementary Fig. S6).

Low correlation was observed among the variables. EFA was not possible for COVID-19 related knowledge. Regression coefficients obtained from each of the 8 COVID-19 related knowledge questions were used to weigh total COVID-19 related knowledge (Supplementary: Tables S1 & S2).

### Association of COVID-19 related knowledge with both fear about SARS-COV-2 infection and severe COVID-19

COVID-19 related knowledge was independently associated with both fear about SARS-COV-2 infection and severe COVID-19, accounting for the variability across country (Tables 3 and 4). The mean for the weighted COVID-19 related knowledge using the coefficients of the logistic regression for fear about SARS-COV-2 infection and fear about severe COVID-19 were 1.012 and 1.258, respectively.

**Table 3:** Association between COVID-19 related knowledge and Fear of SARS-COV-2 infection

Variables	Category	Afraid	Unafraid	*P* Value	Crude OR	95%CI lower	95%CI upper	Adjusted OR^a^	95%CI lower	95%CI upper
Age group	17–19 years	6606	8126	< 0.01	1	ref	Ref	1	ref	ref
	20–22 years	18,161	22,183	0.71	1.007	0.96	1.04	1.03	0.98	1.08
	23-25 years	9534	12,563	0.01	0.93	0.89	0.97	1.01	0.96	1.07
	26–28 years	3257	4050	0.70	0.98	0.93	1.04	1.10	1.02	1.18
	> = 29 years	4823	4860	< 0.01	1.22	1.15	1.28	1.20	1.12	1.28
Sex	Male	9026	16,424	< 0.01	1	ref	Ref	1	Ref	ref
	female	33,071	35,033	< 0.01	1.71	1.66	1·76	1.68	1.63	1.74
	X	284	325	< 0.01	1.59	1.35	1.86	1.45	1.21	1.75
Educational level	Bachelor	27,883	33,093	< 0.01	1	ref	ref	1	ref	Ref
	Master	9365	12,539	< 0.01	0.88	0.85	0.91	0.97	0.93	1.01
	PhD	1746	2146	0.29	0.96	0.90	1.03	0.94	0.86	1.02
	other	2428	3040	0.06	0.94	0.89	1.002	0.96	0.90	1.03
Field of study	Non- health field	32,530	38,912	< 0.01	1	ref	ref	1	Ref	ref
	Health	7975	10,903	< 0.01	0.87	0.84	0.90	0.77	0.74	0.80
Underlying medical condition	None	34,319	45,043	< 0.01	1	ref	ref	1	ref	Ref
	present	6960	5717	< 001	1.59	1.54	1.66	1.53	1.46	1.60
	Prefer not to say	1036	966	< 001	1.40	1.29	1.54	1.53	1.37	1.71
Personal network infection history	Yes	17,123	19,482	< 001	1	ref	ref	1	ref	ref
	No	25,178	32,205	< 001	0.88	0.86	0.91	0.99	0.96	1.03
Govt Timely info.	Strongly agree	4234	6187	< 0.01	ref	ref	ref	1	ref	ref
	agree	14,007	18,941	0.007	1.08	1.03	1.12	1.11	1.05	1.17
	Neither agree nor disagree	8947	10,495	< 0.01	1.24	1.18	1.30	1.21	1.15	1.29
	disagree	9688	10,327	< 0.01	1.37	1.30	1.43	1.34	1.26	1.42
	Strongly agree	4543	4672	< 0.01	1.42	1.34	1.50	1.42	1.32	1.52
Total knowledge	Good	29,576	36,435	0.013	ref	ref	ref	Ref	ref	ref
	Poor	12,790	12,472	0.013	0.96	0.94	0.99	1.05	1.01	1.08
Variables	Mean	β	SD	*P*-value	Crude OR	95%CI lower	95%CI upper	Adjusted OR^a^	95%CI lower	95%CI upper
Risk perception	4.14	0.19	0.003	< 0.01	1.197	1.190	1.20	1.21	1.20	1.22
Alcohol use increase^a^	−0.34	0.09	0.009	< 0.01	1.09	1.03	1.11	1.09	1.07	1.11
Smoking increase^a^	−0.097	−0.03	0.01	< 0.01	0.94	0.92	0.96	0.96	0.95	0.98
Moderate physical activity increase^a^	−0.42	−0.02	0.004	< 0.01	0.94	0.94	0.95	0.97	0.96	0.98
Vigorous physical activity increase^a^	−0.144	− 0.001	0.005	0.79	0.99	0.98	1	0.99	0.98	1.00
Sufficient Financial resources increase^a^	−0.379	− 0.12	0.007	< 0.01	0.86	0.85	0.87	0.88	0.87	0.89
Measurement adherence	8.023	0.16	0.004	< 0.01	1.20	1.19	1.21	1.17	1.16	1.18

^a^Increase– refers to the positive increase in a variable during the COVID-19 minus before the COVID-19 pandemic measurements

**Table 4:** Association between COVID-19 related knowledge and Fear about Severe COVID-19

Variables	Category	Afraid	Unafraid	*P* Value	Crude OR	95%CI lower	95%CI upper	Adjusted OR^a^	95%CI lower	95%CI upper
Age group	17–19 years	7293	7439	0.22	1	ref	ref	1	ref	ref
	20–22 years	18,713	21,631	< 0.01	0.88	0.84	0.91	0.98	0.94	1.03
	23-25 years	9631	12,466	< 0.01	0.78	0.75	0.82	1.00	0.94	1.05
	26–28 years	3239	4068	< 0.01	0.81	0.76	0.85	1.11	1.03	1.19
	> = 29 years	4926	4757	0.03	1.05	1.003	1.11	1.24	1.16	1.33
Sex	Male	9305	16,145	< 0.01	1	ref	ref	1	Ref	Ref
	female	34,216	33,888	< 0.01	1.75	1.70	1.80	1.73	1.67	1.79
	X	281	328	< 0.01	1.48	1.26	1.74	1.40	1.16	1.68
Educational level	Bachelor	29,272	31,704	< 0.01	1	ref	ref	1	ref	Ref
	Master	9483	12,421	< 0.01	0.82	0.80	0.85	0.90	0.87	0.94
	PhD	1690	2202	< 0.01	0.83	0.77	0.88	0.90	0.83	0.98
	other	2446	3022	< 0.01	0.87	0.82	0.92	0.94	0.88	1.01
Field of study	Non- health field	33,534	37,908	< 0.01	1	ref	ref	1	Ref	Ref
	health	8070	10,908	< 0.01	0.84	0.81	0.87	0.80	0.77	0.84
Underlying medical condition	None	34,459	44,903	< 0.01	1	ref	ref	1	ref	Ref
	present	1082	920	< 001	2.38	2.28	2.47	2.50	2.38	2.62
	Prefer not to say	8192	4485	< 001	1.53	1.4	1.67	1.71	1.54	1.91
Personal network infection history	Yes	17,631	18,974	< 001	1	ref	ref	1	ref	ref
	No	26,085	31,298	< 001	0.89	0.87	0.92	1.04	1.01	1.07
Govt Timely info.	Strongly agree	4186	6235		ref	ref	ref	1	ref	ref
	agree	14,341	18,607	< 001	1.08	1.03	1.12	1.12	1.06	1.18
	Neither agree nor disagree	9533	9909	< 001	1.24	1.18	1.30	1.24	1.17	1.32
	disagree	10,015	10,000	< 001	1.37	1.30	1.43	1.28	1.21	1.36
	Strongly agree	4748	4467	< 001	1.42	1.34	1.50	1.33	1.24	1.42
Total knowledge	Good	29,576	36,435	< 0.01	ref	ref	ref	ref	ref	Ref
	Poor	12,790	12,472	< 0.01	1.26	1.22	1.30	1.36	1.31	1.40
Variables	Mean	β	SD	P-value	Crude OR	95%CI lower	95%CI upper	Adjusted OR^a^	95%CI lower	95%CI upper
Risk perception	4.14	0.15	0.003	< 0.01	1.16	1.15	1.17	1.166	1.15	1.17
Alcohol use change^a^	−0.34	0.08	0.009	< 0.01	1.06	1.04	1.08	1.08	1.06	1.10
Smoking increase^a^	−0.097	−0.02	0.01	< 0.01	0.95	0.92	0.96	0.97	0.95	0.99
Moderate physical activity increase^a^	−0.42	−0.01	0.004	< 0.01	0.96	0.95	0.97	0.98	0.97	0.99
Vigorous physical activity increase^a^	−0.144	0.002	0.005	0.55	1.006	0.99	1.01	1.00	0.99	1.01
Sufficient Financial resources increase^a^	−0.379	− 0.11	0.007	< 0.01	0.86	0.85	0.88	0.89	0.88	0.90
Measurement adherence	8.023	0.11	0.004	< 0.01	1.148	1.14	1.15	1.12	1.11	1.13

^a^Increase– refers to the positive increase in a variable during the COVID-19 minus before the COVID-19 pandemic measurements

The odds of being afraid of acquiring SARS-COV-2 infection in those with poor knowledge was 1.05 times the odds of those respondents with good knowledge, accounting for variability across 26 countries. Meanwhile, the odds of being afraid about getting severe COVID-19 in those with poor knowledge was 1.36 times the odds of those respondents with good knowledge.

The country level random effect had a variance of 0.25 and standard deviation of 0.50 for the association between COVID-19 related knowledge deficit and fear about SARS-COV-2 infection (Table 3) while country level random effect had a variance of 0.30 and standard deviation of 0.54 for the association between COVID-19 related knowledge and fear about severe COVID-19 (Table 4).

Field of study was independently associated with both fear of SARS-COV-2 infection and severe COVID-19, accounting for the variability across country. The odds of being afraid about acquiring SARS-COV-2 infection among health field students was 0.77 times the odds of those respondents who were non-health students. Concurrently, the odds of being afraid about getting severe COVID-19 among health field students was 0.80 times the odds of those respondents who were non-health students.

Timely information given by government sources was also independently associated with fear about SARS-COV-2 infection as well as severe COVID-19. The odds of being afraid about acquiring SARS-COV-2 infection among those who strongly disagreed to have received timely information was 1.42 times the odds of those who strongly agreed to have received timely information. Meanwhile, the odds of being afraid about contracting severe COVID-19 among those who strongly disagreed to have received timely information was 1.33 times the odds of those respondents who strongly agreed.

Modifiable risk factors: Smoking and moderate physical activity were independently associated with both fear about SARS-COV-2 infection and severe COVID-19. For a unit increase in average cigarette smoking per day and moderate physical activity (average 30 min. Session/week), the odds of being afraid about acquiring SARS-COV-2 infection decreased in average by 0.96 and 0.95 times, respectively. Simultaneously, for a unit increase in the average smoking per day and moderate physical activity (average 30 min. Session/week), the odds of being afraid about contracting severe COVID-19 decreased in average by 0.97 and 0.98 times, respectively.

Alcohol use was also independently associated with fear about SARS-COV-2 infection as well as severe COVID-19. The odds of being afraid about acquiring SARS-COV-2 infection was 1.09 times higher for a unit increase while the odds of being afraid about contracting severe COVID-19 was 1.08 times higher for a unit increase during the COIVD-19 pandemic.

## Discussion

This study revealed that 62.7% of respondents across the 26 countries were classified as having “good” COVID-19-related knowledge. It further revealed that at country level Romania had the highest proportion of respondents classified as “good” knowledge (89%), while the lowest proportion was seen in Russia (31.8%). Previous studies conducted in the general population and among health care professionals have shown that advanced training and work experience is associated with COVID-19 related knowledge [21–23]. These findings might explain our result, as Romania had the highest proportion of health field students and hence the highest level of COVID-19 related knowledge. Our finding is consistent with a study conducted in India solely among dental students that found adequate COVID-19 related knowledge was 83% [10]. Although the finding in the aforementioned study is higher than the total COVID-19 related knowledge across the 26 countries, it is comparable to countries such as Romania and Portugal which have very high proportion of health students. In contrast, Our findings with regards to COVID-19 related knowledge were much higher compared to a study conducted in Romania (10.8%) among oncology patients [24]. The discrepancy observed between the two studies maybe due to the fact that our study was conducted solely among HE students while the former was conducted with patients where the majority of whom (66.6%) had no formal education. Other reasons for the high percentage of COVID-19 related knowledge in countries such as Romania and Portugal may be related to their early public health responses to curb the spread of the pandemic which may have included governments actively communicating and engaging with those specific communities before introducing a particular measure. This action might have created awareness of COVID-19 early on leading to higher knowledge levels [25]. According to public health measures data from European Union (EU) countries, both Romania and Portugal closed educational institutions early. In comparison the Czech Republic, which had the lowest COVID-19 related knowledge level among EU countries, did not close educational institutions and this may have contributed to low COVID-19 related knowledge [25].

There is limited research about the association of knowledge and fear about COVID-19. Our study revealed that, after adjusting for potential confounders, COVID-19 related knowledge is independently associated with both fear about SARS-COV-2 infection and severe COVID-19. The odds of being afraid about acquiring SARS-COV-2 infection and severe COVID-19 among those who had good knowledge was lower compared to those who had poor knowledge.

Our findings suggest that increasing comprehensive COVID-19-related knowledge, might be associated with both lower fear about severe COVID-19 as well as SARS-COV-2 infection. The reason might be that respondents with more COVID-19 related knowledge will possess a greater sense of control over the pandemic, and thus will not feel threatened enough to fear too much. This is reflected by a study conducted in China which found that those with low self-control were more likely to have exaggerated perception of seriousness to COVID-19 [26]. This finding is also consistent with other variables in our study such as field of study where the odds of being fear about acquiring either SARS-COV-2 or severe COVID-19 among health field students was lower compared to non-health students. The reason behind such finding might be because health students are expected to have more COVID-19 related knowledge and thus high self-control leading to less fear compared to their non-health students counterparts. Another important reason might be related to the infodemic specifically misinformation spread through mainly social media platforms during the pandemic which non-health students are vulnerable to compared to health students [12]. This is especially true among the adolescent age group because they are the major consumers of social media and are exposed to problematic social media use with misinformation as one of its consequences [13].

It is also worth noting that the odds of being afraid of acquiring either SARS-COV-2 infection or severe COVID-19 in those students who strongly agreed receiving timely government information concerning the COVID-19 pandemic was lower, as compared to those who strongly disagreed. This finding is similar to a study conducted in Turkey where satisfaction with specific up-to-date and accurate health information provided was associated with a lower level of anxiety and stress [27]. Our findings, therefore, indicate that factors such as field of study and timely government information that increase COVID-19 related knowledge are associated with lower fear about acquiring SARS-COV-2 infection and severe COVID-19. Additionally, receiving timely information from credible sources such as the government might also be specifically associated with lower fear about acquiring SARS-COV-2 infection and severe COVID-19 as it helps fight the infodemic and decrease the misinformation which usually leads to exaggerated fear [12].

The negative burden of the COVID-19 pandemic especially on the mental health of HE students has been documented [16, 17]. Fear about acquiring severe COVID-19 and SARS-COV-2 infection and lack of sufficient knowledge could further contribute to higher level of depression amongst this population group, which is a leading public health concern.

Increase in smoking was negatively associated with both fear about SARS-COV-2 and severe COVID-19. The myth that smokers are protected against SARS-COV-2 infection has received wide attention on media and has led to some confusion even among the medical community [28]. Thus, erroneous claims communicated about a protective effect of smoking might be the reason behind the peculiar finding in our study. Physical activity particularly increase in moderate physical activity was also negatively associated with both fear about SARS-COV-2 infection and severe COVID-19. Our finding was similar to a study conducted in the United States which found that increase change in physical activity is associated with a reduction in fear about COVID-19, post-traumatic stress disorders and other mental health outcomes due to the COVID-19 pandemic [29]. However, independent association was not observed between vigorous physical activity and fear about SARS-COV-2 infection or severe COVID-19. This might be because the lockdown measures in place were not conducive for performing vigorous physical activity while these measures were suitable for conducting moderate physical activity. Additionally, it is worth noting other studies indicating that the transition to virtual learning environment due to the lock down measures introduced during the pandemic can all in itself further trigger mental health issues [17, 30]. Lastly, increase alcohol use was positively associated with both fear of SARS-COV-2 infection and severe COVID-19. This finding echoes Reynolds et al. [31] study in Ireland where fear during the COVID-19 lockdown period was associated with increased alcohol consumption.

The limitation of this study is that it is cross sectional and does not allow an examination of the possible causal effect of knowledge deficit on fear about SARS-COV-2 infection and severe COVID-19. Additionally, the study used convenience sampling which is not representative of the study population. In addition as the study includes behavioural related questions, social desirability bias is possible as the potential confounders.

The strengths of this study however are: it is a cross-country survey, has a large sample size, examined the random effects in 26 countries and observed the differences between them. In addition it assessed the fear about both SARS-COV-2 infection and Severe COVID-19.

The findings of this study will inform HEIs about the COVID-19 related knowledge level of their students, and provide a basis for action to increase awareness and understanding among the student body. It will also aid to tackle exaggerated fear about severe COVID-19 and SARS-COV-2 infection which can lead to psychological problems such as depression. In addition, by providing targeted and timely interventions to inform, solve COVID-19 knowledge deficit and fight infodemic, will promote psychological well-being and prevent mental health issues in HE students both in the current but also in future pandemics.

Intervention by all stakeholders to tackle fear should not only be implemented by increasing COVID-19 related knowledge level, but also by focusing on timely information through media sources, scaling up intervention on healthy behaviours such as moderate physical activity. More importantly, government bodies in addition to providing timely, up-to-date and accurate information should implement national public health measures that targets both general and specific population groups such as HE students as early as possible, since this action could increase COVID-19 related knowledge, counter the infodemic and reduce fear.

A follow up study is recommended to assess the current level of students’ knowledge and fear 16 months post initial lockdown, as well as such relationship in low income countries. We also recommend to further explore the relationship between physical activity specifically moderate physical activity and fear as it has been found to be associated in our study. Furthermore, the future studies should also assess the causal effect of COVID-19 related knowledge deficit and fear about COVID-19.

## Conclusion

The study explored that there are large differences of COVID-19 related knowledge across countries. Furthermore, the study revealed that total COVID-19 knowledge is independently associated with fear about severe COVID-19 as well as SARS-COV-2 infection after accounting for the variability across 26 countries. Designing targeted interventions early to increase the comprehensive and updated COVID-19 related knowledge should be entertained in order to tackle exaggerated fear and its psychological as well as social complications. Lastly, Our study findings serve as a basis for public health response for both the current and similar future pandemics by highlighting the need for addressing COVID-19 knowledge deficit to fight the infodemic and prevent negative mental health outcomes.

## Supplementary Information

**Additional file 1: Table S1.** COVID-19 related knowledge deficit and fear of SARS-COV2 infection Mixed effect logistic regression (regression coefficient used for COVID-19 related knowledge). **Table S2.** COVID-19 related knowledge deficit and fear of Severe COVID-19 illness Mixed effect logistic regression (regression coefficient used for COVID-19 related knowledge). **Figure S1.** Directed acyclic graph - association between COVID-19 related knowledge deficit and fear of acquiring SARS-COV2 infection. **Figure S2.** Directed acyclic graph - association between COVID-19 related knowledge deficit and fear of acquiring Severe COVID-19. **Figure S3.** Exploring the missing data pattern of missingness and percentage of missingness (R output). **Figure S4.** Exploring Missing data (R output). **Figure S5.** Respondents field of study by country. **Figure S6.** COVID-19 related knowledge by country.

## Data Availability

All data generated or analysed during this study are included in this published article (and its supplementary information files).

## Abbreviations

COVID-19: Coronavirus disease
C-19 ISWS: COVID-19 International students’ well-being study
DAG: Directed acyclic graph
EFA: Explanatory factor analysis
HE: Higher education
HEI: Higher education institutions
MICE: Multiple imputation chained equation
WHO: World Health Organization
SARS-COV-2: Severe acute respiratory syndrome coronavirus 2
