# Supplementary material for: Knowledge deficit and fear of COVID-19 among higher education students during the first wave of the pandemic and implications for public health: a multi-country cross-sectional survey
Source: BMC Public Health. 2022 Jun 7;22:1144. doi: 10.1186/s12889-022-13511-3 (PMC9172614; doi:10.1186/s12889-022-13511-3)
Supplement: Supplementary file 1 — Additional file 1: Table S1. COVID-19 related knowledge deficit and fear of SARS-COV2 infection Mixed effect logistic regression (regression coefficient used for COVID-19 related knowledge). Table S2. COVID-19 related knowledge deficit and fear of Severe COVID-19 illness Mixed effect logistic regression (regression coefficient used for COVID-19 related knowledge). Figure S1. Directed acyclic graph - association between COVID-19 related knowledge deficit and fear of acquiring SARS-COV2 infection. Figure S2. Directed acyclic graph - association between COVID-19 related knowledge deficit and fear of acquiring Severe COVID-19. Figure S3. Exploring the missing data pattern of missingness and percentage of missingness (R output). Figure S4. Exploring Missing data (R output). Figure S5. Respondents field of study by country. Figure S6. COVID-19 related knowledge by country. [file 12889_2022_13511_MOESM1_ESM.docx]

Supplementary Materials

List of Tables

[Table S1: COVID-19 related knowledge deficit and fear of SARS-COV2 infection Mixed effect logistic regression (regression coefficient used for COVID-19 related knowledge) 8](#_Toc82936701)

[Table S2: COVID-19 related knowledge deficit and fear of Severe COVID-19 illness Mixed effect logistic regression (regression coefficient used for COVID-19 related knowledge) 11](#_Toc82936702)

List of Figures

[Figure S1: Directed acyclic graph - association between COVID-19 related knowledge deficit and fear of acquiring SARS-COV2 infection 2](#_Toc82936659)

[Figure S2: Directed acyclic graph - association between COVID-19 related knowledge deficit and fear of acquiring Severe COVID-19 3](#_Toc82936660)

[Figure S3: Exploring the missing data pattern of missingness and percentage of missingness (R output) 4](#_Toc82936661)

[Figure S4: Exploring Missing data (R output) 5](#_Toc82936662)

[Figure S5:. Respondents field of study by country 6](#_Toc82936663)

[Figure S6: COVID-19 related knowledge by country 7](#_Toc82936664)


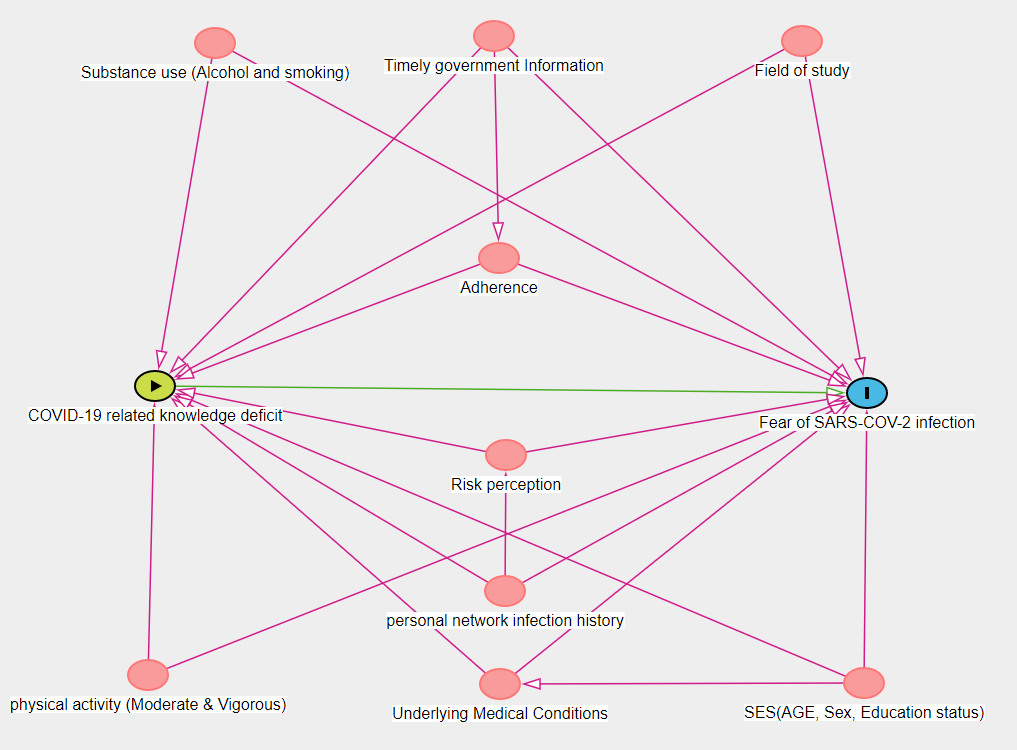


Figure S1: Directed acyclic graph - Association between COVID-19 knowledge deficit and fear of acquiring SARS-COV2 infection


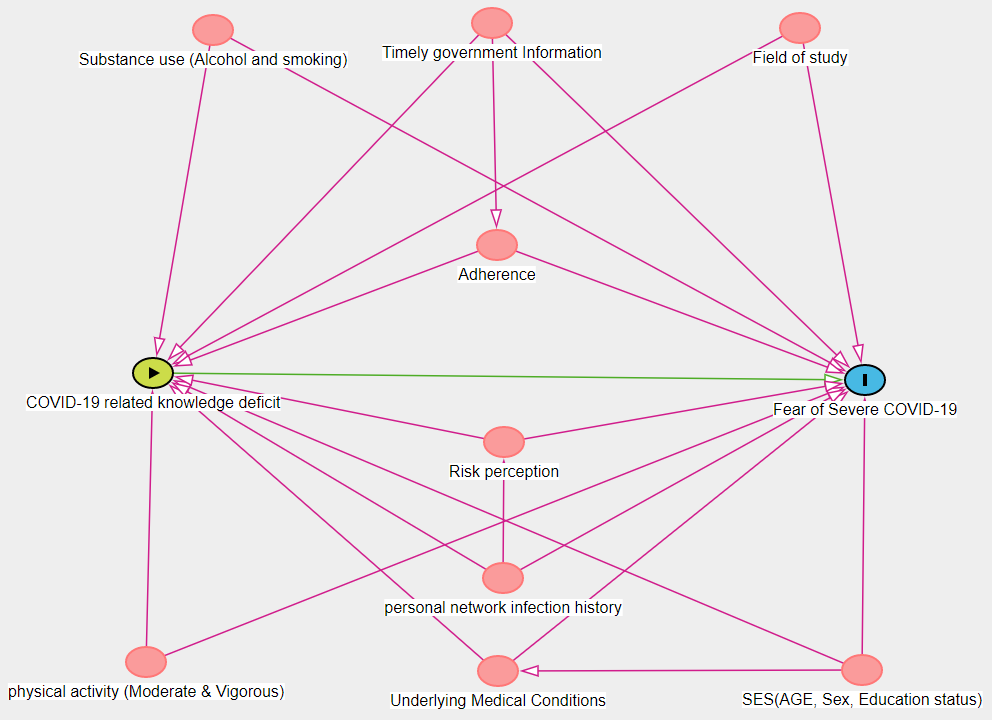


Figure S2: :Directed acyclic graph - association between COVID-19 related knowledge deficit and fear of acquiring Severe COVID-19


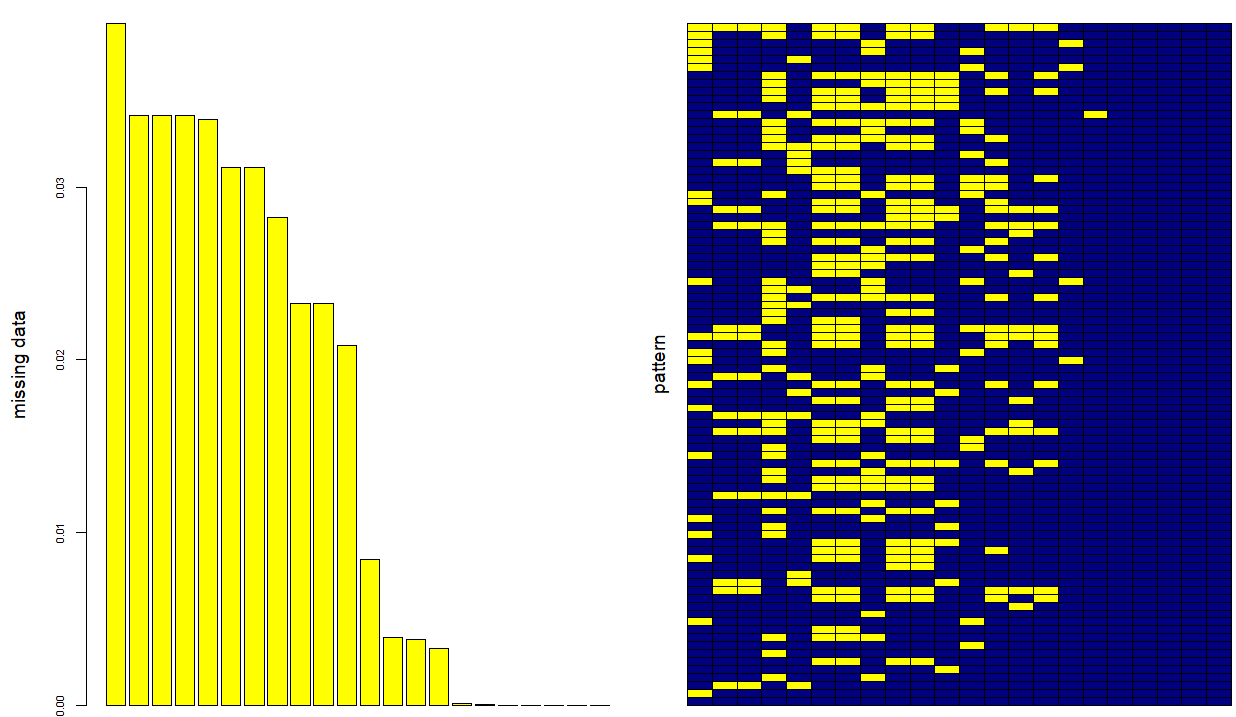


Figure S3: Exploring the missing data pattern of missingness and percentage of missingness (R output)


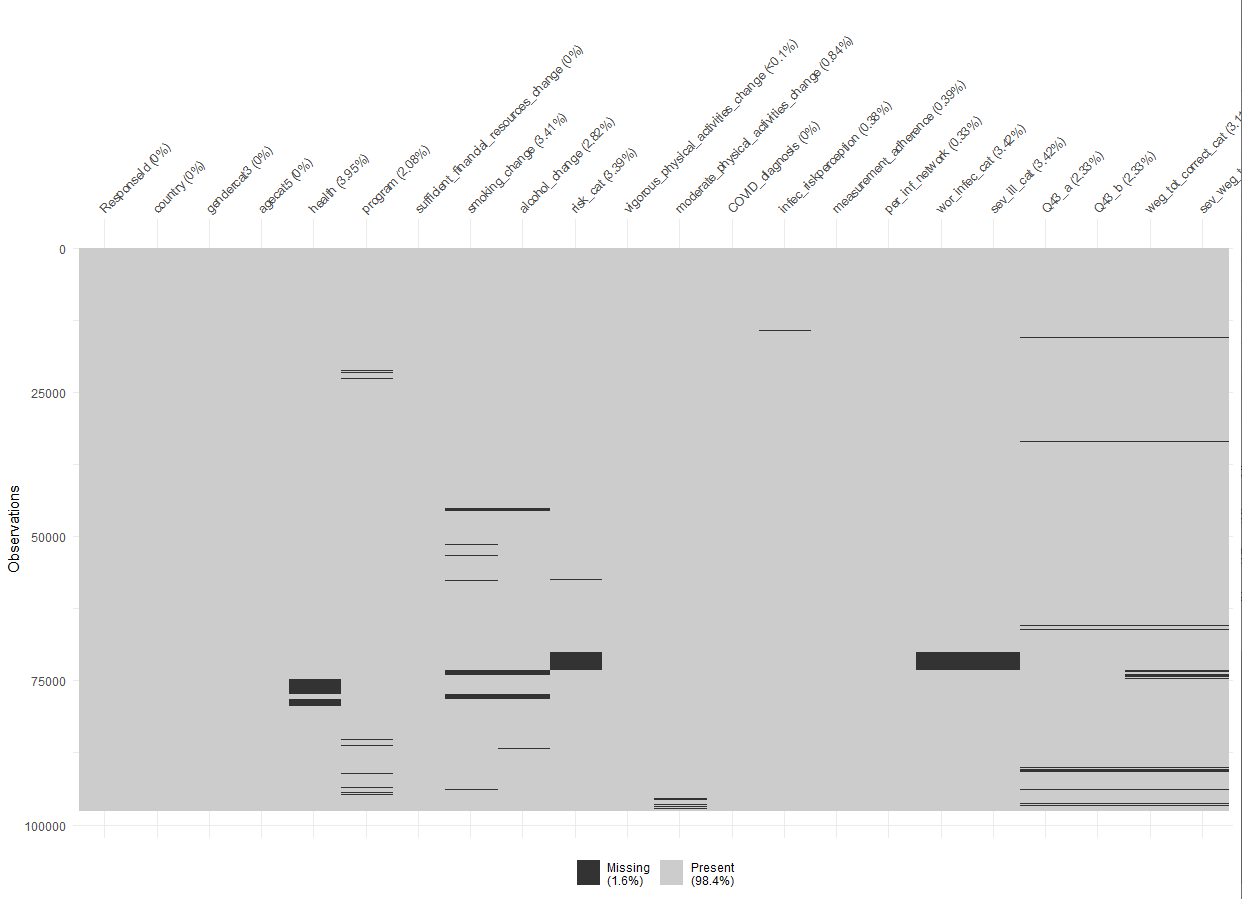


Figure S4: Exploring Missing data (R output)

* Q43_a = “Did The government provided information concerning the COVID-19 outbreak on time”

* Q43_b= “The government provided comprehensive information concerning the COVID-19 outbreak”

* risk_cat= “Do you have any of the following underlying conditions?”

*per_inf_network= “Do you know anyone in your personal network that was or currently is infected with COVID-19?”

*wor_infec_cat= “How worried are you that you will get infected with COVID-19?”

* Sev_ill_cat = “How worried are you that you will get severely ill from a COVID-19 infection?

Figure S5:. Respondents field of study by country

Figure S6: COVID-19 related knowledge by country

Table S1: COVID-19 related knowledge deficit and fear of SARS-COV-2 infection Mixed effect logistic regression (regression coefficient used for COIVD-19 related knowledge)

|  |  | Afraid | Unafraid | P Value | Crude OR | 95%CI lower | 95%CI  upper | | Adjusted OR* | 95%CI lower | 95%CI upper |
| --- | --- | --- | --- | --- | --- | --- | --- | --- | --- | --- | --- |
| Age group  (***) | 17-19 years | 6606 | 8126 | <0.01 | 1*** | ref | ref | | 1 | ref | ref |
|  | 20-22 years | 18161 | 22183 | 0.71 | 1.007 | 0.96 | 1.04 | | 0.97 | 0.92 | 1.01 |
|  | 23-25years | 9534 | 12563 | 0.01 | 0.93 | 0.89 | 0.97 | | 0.91 | 0.86 | 0.96 |
|  | 26-28 years | 3257 | 4050 | 0.70 | 0.98 | 0.93 | 1.04 | | 0.96 | 0.90 | 1.03 |
|  | >= 29 years | 4823 | 4860 | <0.01 | 1.22 | 1.15 | 1.28 | | 1.15 | 1.08 | 1.23 |
| Sex  (***) | Male | 9026 | 16424 | <0.01 | 1 | ref | ref | | 1 | Ref | ref |
|  | female | 33071 | 35033 | <0.01 | 1.71 | 1.66 | 1·76 | | 1.62 | 1.57 | 1.68 |
|  | X | 284 | 325 | <0.01 | 1.59 | 1.35 | 1.86 | | 1.33 | 1.10 | 1.59 |
| Educational level  (***) | Bachelor | 27883 | 33093 | <0.01 | 1 | ref | ref | | 1 | ref | Ref |
|  | Master | 9365 | 12539 | <0.01 | 0.88 | 0.85 | 0.91 | | 0.91 | 0.87 | 0.94 |
|  | PhD | 1746 | 2146 | 0.29 | 0.96 | 0.90 | 1.03 | | 0.94 | 0.87 | 1.02 |
|  | other | 2428 | 3040 | 0.06 | 0.94 | 0.89 | 1.002 | | 0.95 | 0.89 | 1.02 |
| Field of study  (***) | Non- health field | 32530 | 38912 | <0.01 | 1 | ref | ref | | 1 | Ref | ref |
|  | health | 7975 | 10903 | <0.01 | 0.87 | 0.84 | 0.90 | | 0.76 | 0.74 | 0.79 |
| Underlying medical condition | None | 34319 | 45043 | <0.01 | 1 | ref | ref | | 1 | ref | Ref |
|  | present | 6960 | 5717 | <001 | 1.59 | 1.54 | 1.66 | | 1.41 | 1.35 | 1.48 |
|  | Prefer not to say | 1036 | 966 | <001 | 1.40 | 1.29 | 1.54 | | 1.40 | 1.26 | 1.56 |
| Govt Timely info. | Strongly agree | 4234 | 6187 | <0.01 | ref | ref | ref | | 1 | ref | ref |
|  | agree | 14007 | 18941 | 0.007 | 1.08 | 1.03 | | 1.12 | 1.16 | 1.10 | 1.22 |
|  | Neither agree nor disagree | 8947 | 10495 | <0.01 | 1.24 | 1.18 | | 1.30 | 1.38 | 1.31 | 1.46 |
|  | disagree | 9688 | 10327 | <0.01 | 1.37 | 1.30 | | 1.43 | 1.52 | 1.44 | 1.61 |
|  | Strongly agree | 4543 | 4672 | <0.01 | 1.42 | 1.34 | | 1.50 | 1.72 | 1.61 | 1.84 |
| Knowledge  question a | Incorrect | 17108 | 19417 | <0.01 | ref | ref | | ref | 1 | ref | ref |
|  | Correct | 24243 | 31179 | <0.01 | 0.88 | 0.85 | | 0.90 | 0.94 | 0.91 | 0.98 |
| Knowledge  question b | Incorrect | 28061 | 37848 | <0.01 | ref | ref | | ref | 1 | ref | ref |
|  | Correct | 13142 | 12646 | <0.01 | 1.40 | 1.36 | | 1.44 | 0.76 | 0.73 | 0.78 |
| Knowledge  question c | Incorrect | 6335 | 5168 | <0.01 | ref | ref | | ref | 1 | ref | ref |
|  | Correct | 35089 | 45475 | <0.01 | 0.62 | 0.60 | | 0.65 | 0.54 | 0.51 | 0.58 |
| Knowledge  question d | Incorrect | 11615 | 16745 | <0.01 | ref | ref | | ref | 1 | ref | ref |
|  | Correct | 29678 | 33811 | <0.01 | 1.26 | 1.23 | | 1.30 | 0.79 | 0.74 | 0.84 |
| Knowledge  question e | Incorrect | 14791 | 19731 | <0.01 | ref | ref | | ref | 1 | ref | ref |
|  | Correct | 26519 | 30842 | <0.01 | 1.14 | 1.11 | | 1.17 | 0.88 | 0.84 | 0.92 |
| Knowledge  question f | Incorrect | 1485 | 1700 | 0.01 | ref | ref | | ref | 1 | ref | ref |
|  | Correct | 39965 | 48957 | 0.06 | 0.93 | 0.87 | | 1.00 | 1.21 | 1.05 | 1.40 |
| Knowledge  question g | Incorrect | 7848 | 8824 | <0.01 | ref | ref | | ref | 1 | ref | ref |
|  | Correct | 33468 | 41755 | <0.01 | 0.90 | 0.87 | | 0.93 | 1.29 | 1.22 | 1.37 |
| Knowledge  question h | Incorrect  (True) | 1307 | 2113 | <0.01 | ref | ref | | ref | 1 | ref | ref |
|  | Correct (False) | 40144 | 48538 | <0.01 | 1.33 | 1.24 | | 1.43 | 1.32 | 1.16 | 1.50 |
| Variables | Mean | β | SD | P-value | Crude OR | 95%CI lower | | 95%CI upper | Adjusted OR* | 95%CI lower | 95%CI upper |
| Risk perception | 4.14 | 0.18 | 0.002 | <0.01 | 1.197 | 1.190 | | 1.20 | 1.20 | 1.19 | 1.21 |
| Alcohol use | -0.34 | 0.08 | 0.007 | <0.01 | 1.09 | 1.03 | | 1.11 | 1.08 | 1.06 | 1.10 |
| Smoking | -0.097 | -0.05 | 0.008 | <0.01 | 0.94 | 0.92 | | 0.96 | 0.93 | 0.91 | 0.95 |
| Moderate physical activity change | -0.42 | -0.05 | 0.003 | <0.01 | 0.94 | 0.79 | | 0.94 | 0.94 | 0.93 | 0.95 |
| Vigorous physical activity change | -0.144 | -.005 | 0.004 | 0.147 | 0.99 | 0.98 | | 1 | 1.01 | 1.00 | 1.02 |
| Sufficient  Financial resources | -0.379 | -0.14 | 0.006 | <0.01 | 0.86 | 0.85 | | 0.87 | 0.89 | 0.87 | 0.90 |
| Measurement adherance | 8.023 | 0.18 | 0.003 | <0.01 | 1.20 | 1.19 | | 1.21 | 1.20 | 1.19 | 1.21 |

Table S2: : COVID-19 related knowledge deficit and fear of Severe COVID-19 illness Mixed effect logistic regression (regression coefficient used for COVID-19 related knowledge)

|  |  | Afraid | Unafraid | P Value | Crude OR | 95%CI lower | 95%CI  upper | | Adjusted OR* | 95%CI lower | 95%CI upper |
| --- | --- | --- | --- | --- | --- | --- | --- | --- | --- | --- | --- |
| Age group  (***) | 17-19 years | 7293 | 7439 | 0.22 | 1 | ref | ref | | 1 | ref | ref |
|  | 20-22 years | 18713 | 21631 | <0.01 | 0.88 | 0.84 | 0.91 | | 0.86 | 0.83 | 0.90 |
|  | 23-25years | 9631 | 12466 | <0.01 | 0.78 | 0.75 | 0.82 | | 0.80 | 0.76 | 0.85 |
|  | 26-28 years | 3239 | 4068 | <0.01 | 0.81 | 0.76 | 0.85 | | 0.84 | 0.79 | 0.90 |
|  | >= 29 years | 4926 | 4757 | 0.03 | 1.05 | 1.003 | 1.11 | | 1.04 | 0.98 | 1.11 |
| Sex  (***) | Male | 9305 | 16145 | <0.01 | 1 | ref | ref | | 1 | Ref | ref |
|  | female | 34216 | 33888 | <0.01 | 1.75 | 1.70 | 1.80 | | 1.66 | 1.61 | 1.72 |
|  | X | 281 | 328 | <0.01 | 1.48 | 1.26 | 1.74 | | 1.27 | 1.06 | 1.52 |
| Educational level  (***) | Bachelor | 29272 | 31704 | <0.01 | 1 | ref | ref | | 1 | ref | Ref |
|  | Master | 9483 | 12421 | <0.01 | 0.82 | 0.80 | 0.85 | | 0.88 | 0.84 | 0.91 |
|  | PhD | 1690 | 2202 | <0.01 | 0.83 | 0.77 | 0.88 | | 0.85 | 0.79 | 0.92 |
|  | other | 2446 | 3022 | <0.01 | 0.87 | 0.82 | 0.92 | | 0.88 | 0.82 | 0.94 |
| Field of study  (***) | Non- health field | 33534 | 37908 | <0.01 | 1 | ref | ref | | 1 | Ref | ref |
|  | health | 8070 | 10908 | <0.01 | 0.84 | 0.81 | 0.87 | | 0.77 | 0.74 | 0.80 |
| Underlying medical condition | None | 34459 | 44903 | <0.01 | 1 | ref | ref | | 1 | ref | Ref |
|  | present | 1082 | 920 | <001 | 2.38 | 2.28 | 2.47 | | 2.29 | 2.19 | 2.40 |
|  | Prefer not to say | 8192 | 4485 | <001 | 1.53 | 1.4 | 1.67 | | 1.51 | 1.36 | 1.58 |
| Govt Timely info. | Strongly agree | 4186 | 6235 | <001 | ref | ref | ref | | 1 | ref | ref |
|  | agree | 14341 | 18607 | <001 | 1.08 | 1.03 | | 1.12 | 1.21 | 1.15 | 1.27 |
|  | Neither agree nor disagree | 9533 | 9909 | <001 | 1.24 | 1.18 | | 1.30 | 1.51 | 1.42 | 1.59 |
|  | disagree | 10015 | 10000 | <001 | 1.37 | 1.30 | | 1.43 | 1.58 | 1.49 | 1.67 |
|  | Strongly agree | 4748 | 4467 | <001 | 1.42 | 1.34 | | 1.50 | 1.76 | 1.64 | 1.88 |
| Knowledge  question a | Incorrect  (True) | 9285 | 9590 | 0.02 | ref | ref | | ref | 1 | ref | ref |
|  | Correct (False) | 24919 | 30503 | <0.01 | 0.84 | 0.81 | | 0.87 | 0.94 | 0.91 | 0.98 |
|  | Don’t Know | 8554 | 9096 | 0.16 | 0.97 | 0.93 | | 1.01 | 1.008 | 0.96 | 1.05 |
| Knowledge  question b | Incorrect  (True) | 13329 | 12459 | <0.01 | ref | ref | | ref | 1 | ref | ref |
|  | Correct (False) | 18773 | 24205 | <0.01 | 0.72 | 0.70 | | 0.74 | 0.79 | 0.76 | 0.82 |
|  | Don’t Know | 10516 | 12415 | <0.01 | 0.79 | 0.76 | | 0.82 | 0.81 | 0.78 | 0.85 |
| Knowledge  question c | Incorrect  (True) | 4271 | 2606 | 0.01 | ref | ref | | ref | 1 | ref | ref |
|  | Correct (False) | 35923 | 44641 | 0.01 | 0.49 | 0.46 | | 0.51 | 0.45 | 0.42 | 0.48 |
|  | Don’t Know | 2634 | 1992 | 0.01 | 0.80 | 0.74 | | 0.87 | 0.80 | 0.73 | 0.88 |
| Knowledge  question d | Correct  (True) | 30116 | 33373 | <0.01 | ref | ref | | ref | 1 | ref | ref |
|  | Incorrect (False) | 2608 | 3393 | <0.01 | 0.85 | 0.80 | | 0.89 | 0.87 | 0.82 | 0.93 |
|  | Don’t Know | 9978 | 12381 | <0.01 | 0.89 | 0.86 | | 0.92 | 0.84 | 0.81 | 0.87 |
| Knowledge  question e | Correct  (True) | 26749 | 30612 | <0.01 | ref | ref | | ref | 1 | ref | ref |
|  | Incorrect (False) | 5152 | 6306 | <0.01 | 0.93 | 0.89 | | 0.97 | 0.99 | 0.95 | 1.04 |
|  | Don’t Know | 10814 | 12250 | 0.51 | 1.01 | 0.97 | | 1.04 | 1.03 | 0.99 | 1.07 |
| Knowledge  question f | Correct  (True) | 41284 | 47638 | <0.01 | ref | ref | | ref | 1 | ref | ref |
|  | Incorrect (False) | 603 | 572 | <0.01 | 1.21 | 1.08 | | 1.36 | 1.36 | 1.18 | 1.56 |
|  | Don’t Know | 964 | 1046 | 0.17 | 1.06 | 0.97 | | 1.16 | 1.09 | 0.98 | 1.23 |
| Knowledge  question g | Correct  (True) | 34509 | 40714 | <0.01 | ref | ref | | ref | 1 | ref | ref |
|  | Incorrect (False) | 3628 | 3548 | <0.01 | 1.20 | 1.14 | | 1.26 | 1.22 | 1.16 | 1.30 |
|  | Don’t Know | 4591 | 4905 | <0.01 | 1.10 | 1.05 | | 1.15 | 1.11 | 1.06 | 1.17 |
| Knowledge  question h | Incorrect  (True) | 618 | 1049 | <0.01 | ref | ref | | ref | 1 | ref | ref |
|  | Correct (False) | 41512 | 47170 | <0.01 | 1.49 | 1.35 | | 1.65 | 1.51 | 1.32 | 1.72 |
|  | Don’t Know | 724 | 1029 | 0.01 | 1.19 | 1.04 | | 1.37 | 1.00 9 | 0.84 | 1.20 |
| Variables | β | SD | P-value | Crude OR | 95%CI lower | 95%CI upper | | Adjusted OR* | 95%CI lower | 95%CI upper |  |
| Risk perception | 0.18 | 0.002 | <0.01 | 1.197 | 1.190 | 1.20 | | 1.21 | 1.20 | 1.22 |  |
| Alcohol use | 0.08 | 0.007 | <0.01 | 1.09 | 1.03 | 1.11 | | 1.09 | 1.07 | 1.11 |  |
| Smoking | -0.05 | 0.008 | <0.01 | 0.94 | 0.92 | 0.96 | | 0.96 | 0.95 | 0.98 |  |
| Moderate physical activity change | -0.05 | 0.003 | <0.01 | 0.94 | 0.79 | 0.94 | | 0.97 | 0.96 | 0.98 |  |
| Vigorous physical activity change | -.005 | 0.004 | 0.147 | 0.99 | 0.98 | 1 | | 0.99 | 0.98 | 1.00 |  |
| Sufficient  Financial resources | -0.14 | 0.006 | <0.01 | 0.86 | 0.85 | 0.87 | | 0.88 | 0.87 | 0.90 |  |
| Measurement adherance | 0.18 | 0.003 | <0.01 | 1.20 | 1.19 | 1.21 | | 1.17 | 1.16 | 1.18 |  |
